# Over-the-scope clip for postsurgical anastomotic dehiscence and bleeding

**DOI:** 10.1016/j.vgie.2024.09.010

**Published:** 2024-09-12

**Authors:** Eduardo Rodríguez-Hernández, Masayoshi Yamada, Takayuki Yamazaki, Shunsuke Tsukamoto, Yutaka Saito

**Affiliations:** 1 Endoscopy Division, National Cancer Center Hospital, Tokyo, Japan; 2 Department of Genetic Medicine and Services, National Cancer Center Hospital, Tokyo, Japan; 3 Department of Colorectal Surgery, National Cancer Center Hospital, Tokyo, Japan

## Introduction

**Figure 1:**
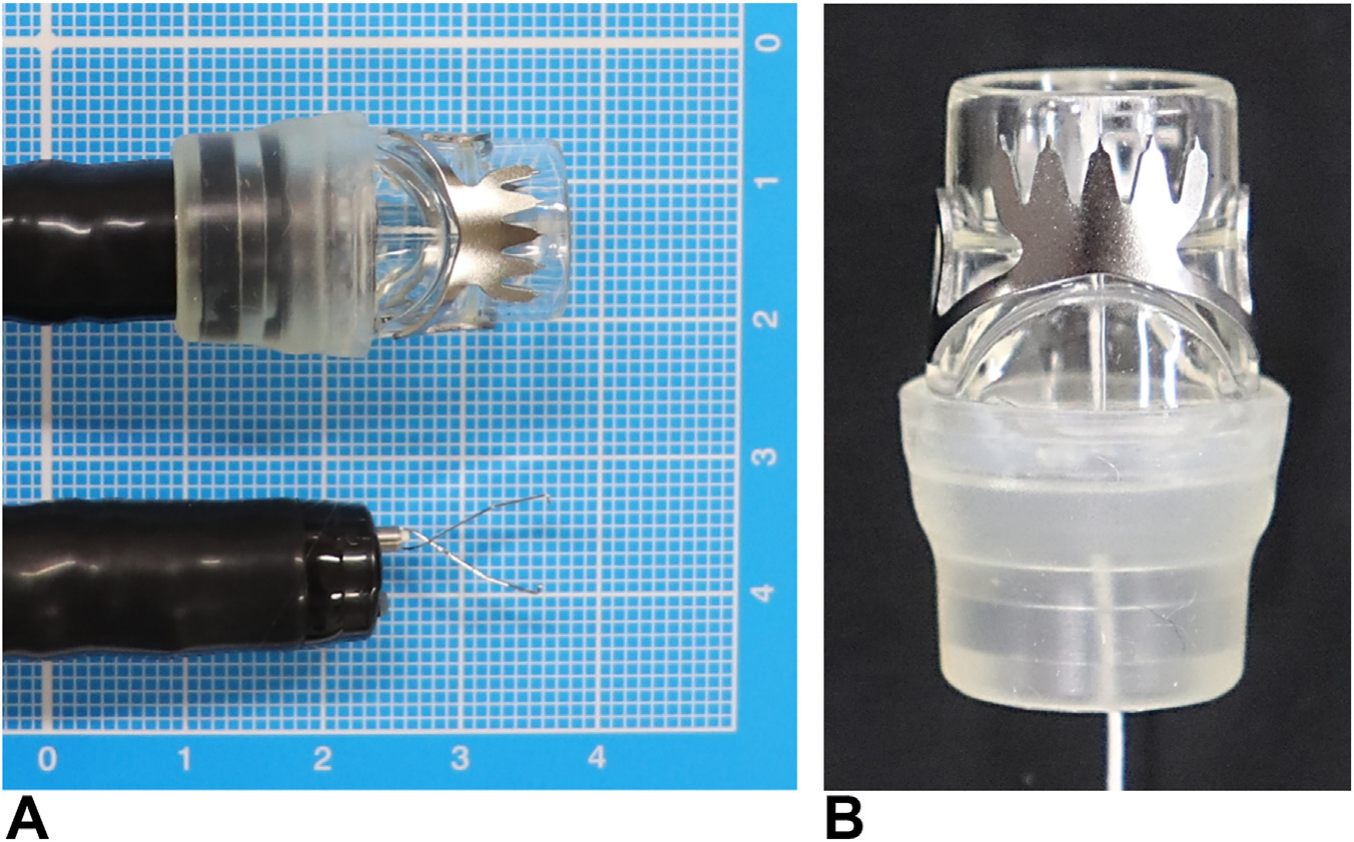
**A,** Comparative photograph of the over-the-scope clip and a conventional clip. **B,** Enlarged photograph of the over-the-scope clip, type T (“traumatic”), showing its serrated edges that ensure secure anchorage after deployment.

Postsurgical anastomotic dehiscence and bleeding are commonly managed with conservative approaches, endoscopic treatment, or surgical reintervention.^1^ However, endoscopic management has become increasingly popular as a less-invasive option in selected cases.^2^ The over-the-scope clip (OTSC) is now a crucial tool in endoscopy units, offering advantages over traditional clips. It effectively closes defects larger than 1 cm (Fig. 1A) and provides strong tissue approximation without causing ischemia or laceration (Fig. 1B).^3^ Originally used for acute iatrogenic perforations, GI bleeding, and postsurgical fistulas, the applications of the OTSC have expanded to include closing PEG tube sites, managing adverse events of endoscopic submucosal dissection, and treating colonic diverticular bleeding.^2^^,^^4^ We present the case of a patient who was successfully treated with the OTSC for dehiscence and bleeding from a postsurgical anastomosis.

## Case presentation

**Figure 2:**
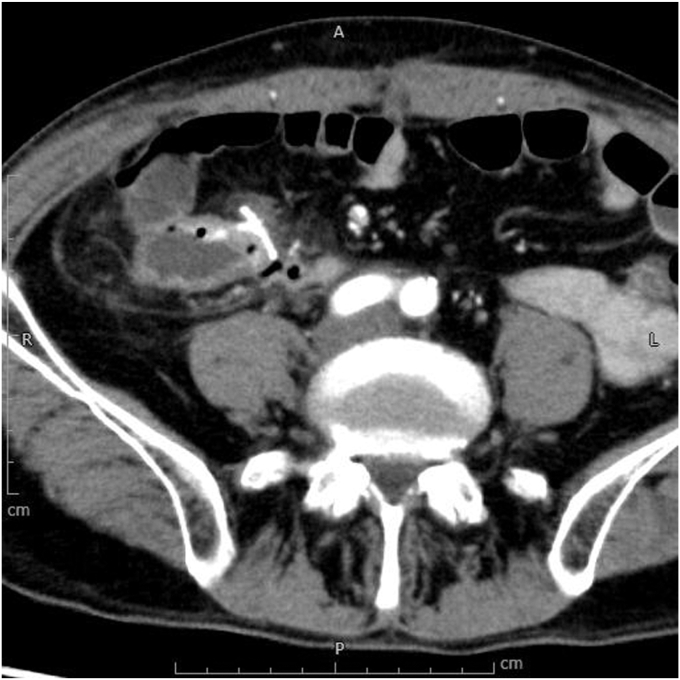
Urgent contrast-enhanced CT scan demonstrating mucosal enhancement on both sides of the ileocolonic anastomosis, along with thickening of the surrounding adipose tissue.

**Figure 3:**
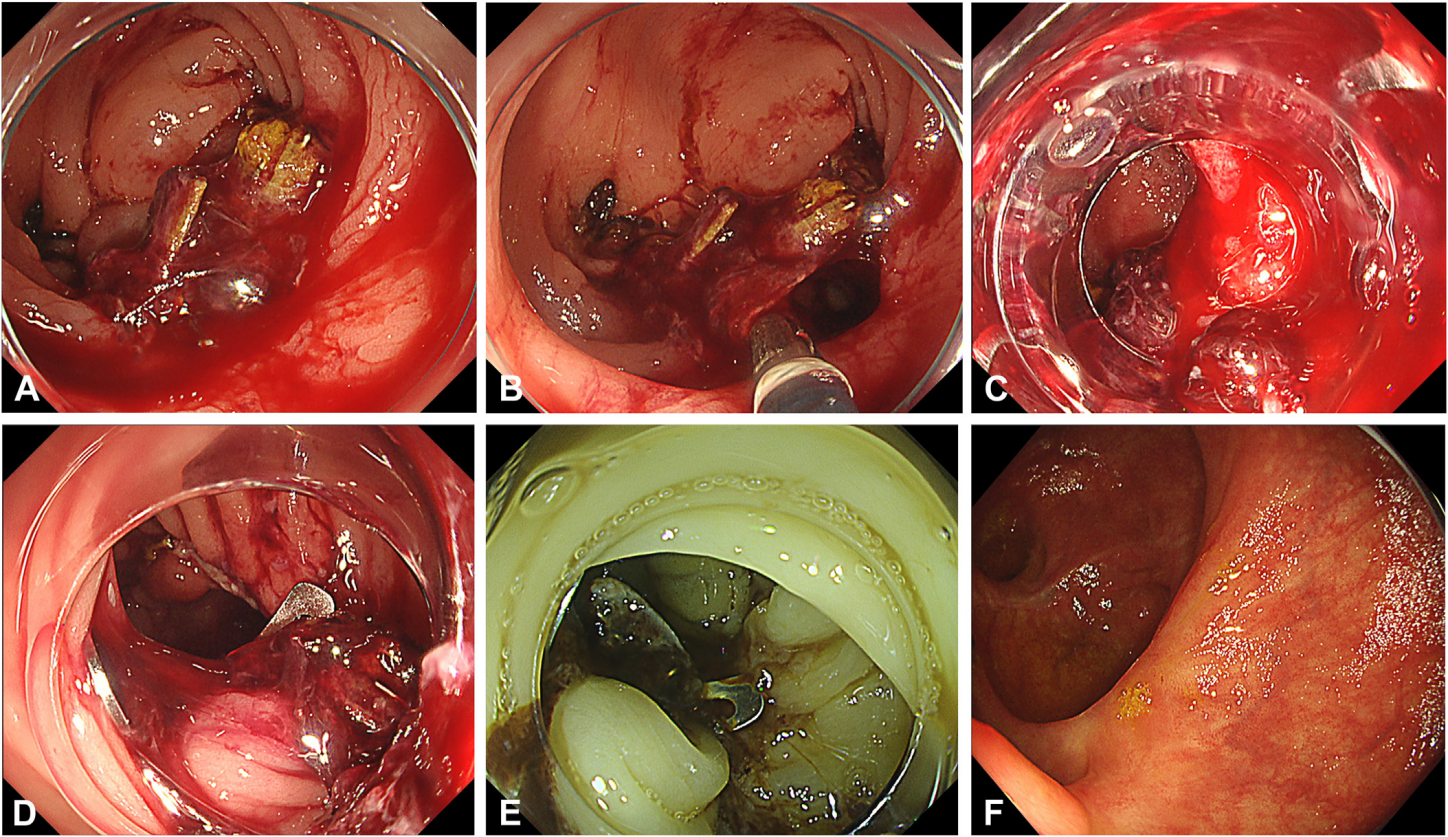
**A,** Adherent clot, granulation tissue, and surgical staples with arterial hemorrhage underneath at the 5-o'clock position in the ileocolonic anastomosis. **B,** Use of grasping forceps revealing an anastomotic dehiscence. **C,** Endoscopic view before placement of the over-the-scope clip. **D,** Over-the-scope clip deployed across the anastomotic line. **E,** Red dichromatic imaging mode after placement of the over-the-scope clip and 2 conventional clips, showing no active bleeding. **F,** Complete anastomotic healing after 12 months.

A 60-year-old male patient with stage T3N0M0 colon cancer underwent a right hemicolectomy. Ten days after surgery, he developed hematochezia, accompanied by tachycardia at 102 beats per minute and a hemoglobin decrease of more than 3 g/dL, reaching 7.9 g/dL. A contrast-enhanced CT scan performed urgently demonstrated mucosal enhancement on both sides of the ileocolonic anastomosis, along with thickening of the surrounding adipose tissue (Fig. 2). The patient received an immediate transfusion of 2 units of blood and was urgently referred for colonoscopy (Video 1, available online at www.videogie.org), which revealed significant bleeding at the anastomotic site. During the inspection, a blood clot, granulation tissue, and arterial hemorrhage were noted at the 5-o'clock position of the anastomotic line (Fig. 3A). A forceps grasper was used to clear the debris, revealing a dehiscence of approximately 1 cm (Fig. 3B). Considering the size of the defect, we opted to use an OTSC. After assembling and positioning the device (Fig. 3C), specialized grasping forceps were used to grasp both the ileal and colonic sides of the anastomosis. These were pulled together into the cap, suctioned, and then the OTSC was released (Fig. 3D), successfully closing the dehiscence and controlling the bleeding. After this, 2 additional conventional clips were placed to the right of the OTSC to optimize the treatment. Red dichromatic imaging confirmed the cessation of bleeding (Fig. 3E). A hemostatic gel (a synthetic self-assembling peptide) was applied for additional support. After the treatment, the patient showed significant hemodynamic improvement, with no recurrence of bleeding. Follow-up endoscopy at 12 months confirmed complete anastomotic healing (Fig. 3F).

## Discussion

Since its introduction in 2010, the OTSC has revolutionized endoscopic interventions, replacing traditional surgical approaches.^2^ In our case, major surgical reintervention was avoided 10 days after a right hemicolectomy. We believe that the OTSC is one of the few closure methods that offers substantial closure force and resistance to deformation. However, it has limitations. First, it requires the withdrawal and reinsertion of the colonoscope with the OTSC, which can be challenging in a tortuous colon or the right side of the colon. In addition, the quick-release mechanism does not allow for gradual adjustment, increasing the risk of improper placement and potential damage to adjacent organs^5^ or critical blood vessels.^6^ The clip, whether as the result of an error in deployment or spontaneous dislodgement, also may cause intestinal obstruction in areas of stenosis or at the anal canal.^7^ Nevertheless, with careful insertion and cautious use, the OTSC is a safe device, with an adverse event rate of 1.7% as reported.^8^ Although it is more expensive than conventional clips, its overall cost-effectiveness may be favorable, considering its potential to reduce the need for surgical reintervention and the associated morbidity.^9^ In conclusion, the OTSC effectively managed postsurgical anastomotic dehiscence and bleeding, avoiding major surgical reintervention and highlighting its value as a therapeutic tool in endoscopy units.

## Patient consent

The patient in this article has given written informed consent to publication of their case details.

## Disclosure

All authors disclosed no financial relationships.

## Supplementary data

Video 1Demonstration of the over-the-scope clip for the treatment of postsurgical anastomotic dehiscence and bleeding.
